# Effects of a spice-blended honey muffin on salivary inflammation markers in adults with obesity: a feasibility trial

**DOI:** 10.1080/07853890.2023.2245325

**Published:** 2023-08-11

**Authors:** Sofia Acevedo, Jeanette M. Andrade

**Affiliations:** University of FL Food Science and Human Nutrition Department, Gainesville, FL, USA

**Keywords:** Chronic inflammation, turmeric, IL-6, cRP, obesity

## Abstract

**Background:**

Obesity is considered a low-grade chronically inflamed state that contributes to communicable chronic diseases. This inflammation may be modulated by consuming spices like turmeric daily. However, few studies have looked at the inclusion of spice within whole foods.

**Objective:**

The purpose of this feasibility pre/posttrial was to assess the influence of turmeric in a muffin on salivary IL-6 and CRP in adults who were obese.

**Methods:**

Participants consumed one, 60-gram muffin containing 3 g turmeric for 10 days. Participants provided a urinary sample at baseline, a 2-ml saliva sample, and a 30-day food frequency and spice consumption questionnaire at baseline and post-trial. A one-sample t-test was conducted using SAS v 9.4 with significance determined at *p* < 0.05.

**Results:**

A total of 14 participants, average BMI of 32.16 kg/m^2^ with 10 identifying as female, completed the trial after 5 dropped due to various reasons. The visit lengths and collection of data with participants adhering to the instructions were deemed a success. There was a significant decrease in salivary IL-6 (*p* = 0.03) but no statistical difference in salivary CRP (*p* = 0.46). Participants consumed fruits and vegetables at least once daily, chicken and eggs 5–6 times per week, and beef, pork, and fish at least once per week. Participants consumed chili pepper, garlic, cinnamon, cilantro, and ginger at least once per week. No changes were observed in dietary/spice habits during this trial.

**Conclusion:**

The feasibility pre/post study revealed that consumption of a muffin with turmeric reduced at least salivary IL-6 in 10 days. Modifications to the study design such as lengthier trial time to assess the impact of this muffin on CRP is necessary prior to implementing larger-scale randomized control trials.

## KEY MESSAGES

- Chronic inflammation in obese patients can be modulated with the use of spices.
- The consumption of a spiced muffin with turmeric (2mg) reduced salivary IL-6 in 10 days (*p* = 0.03).

## Introduction

Obesity, defined as having a body mass index over 30 kg/m^2^, is a public health concern across the world [1]. The world health organization states that 42% of the United States population is classified as obese, which has almost tripled since 1975. Obesity contributes to decreased life expectancy and increased risk of non-communicable diseases like diabetes, chronic kidney disease (CKD), and cardiovascular [2].

Obesity is associated with high levels of inflammation which can be measured through interleukin-6 (IL-6) and C-reactive protein (CRP). Interleukins are a group of cytokines mainly expressed by leukocytes in the inflammation pathway [3]. IL-6 is a specific type of interleukin receptor and a relevant biomarker for metabolic, regenerative, and neural inflammation processes [4]. Commonly, IL-6 is measured through plasma or serum, however, IL-6 can also be measured through saliva. Measuring IL-6 through saliva is valid and may be a simpler and affordable way to detect inflammation [5]. CRP is an acute phase protein whose production is stimulated by IL-6 and secreted by the liver [6,7]. Elevated CRP has been associated with an increased risk of cardiovascular disease, cardiovascular morbidity, and mortality risk in those with chronic kidney disease as well as those suffering from comorbidities related to inflammation [6,8]. CRP has strong proinflammatory activity and during inflammation it binds to damaged and necrotic cells, promoting phagocytosis and helping maintain inflammation [6–8]. Just like IL-6, CRP can be measured through saliva, which allows for inexpensive testing [5].

Studies conducted in adults with various chronic diseases indicate that dietary changes such as incorporating spices into dishes can have a positive impact in reducing inflammation [9–16]. Spices are considered natural plant products that have been used for their medicinal qualities that dates back centuries [17]. More recently, several of these spices, including turmeric, have been shown to reduce inflammation and improve other health outcomes present in chronic diseases such as arthritis, cardiovascular disease, diabetes, kidney disease and obesity [14,18–20]. For instance, a crossover randomized controlled trial with overweight and obese males (*n* = 12) found that a high-fat, high carbohydrate meal containing 6 g of spice blend attenuated the inflammatory effects of the meal by reducing post-prandial IL-1 [19].

More specifically, pre-clinical and clinical studies have been looking at turmeric to reduce inflammation [21–25]. Turmeric is native to and commercially grown in Southeast Asian and is used in various ethnic cuisines. The most active component of turmeric is curcumin, which makes up 2 to 5% of the spice and is considered the component that reduces inflammation [26]. The few clinical studies that have been conducted to determine the relationship between inflammation markers and curcumin have relied on supplements or orally administered curcumin [20,27–29]. Results from these studies have shown that the bioavailability of dietary supplements may not be as high as the consumption of the food or spice directly through meals that include other components that can increase turmeric bioavailability [29,30]. Adding spices to meals can benefit the bioavailability of these chemicals and ease of consumption. For example, the combination of the turmeric spice with the oil in most baked goods can improve bioavailability by up-regulation of lipid absorbent enzymes [29]. The use of whole foods to test the benefits of anti-inflammatory properties of curcumin might yield more beneficial results as these findings would be a more affordable option and easier to incorporate into current dietary habits. Thus, the purpose of this pre/post feasibility trial was to assess the influence of turmeric in a muffin on salivary IL-6 and CRP in adults who were overweight/obese. The feasibility components evaluated included participant recruitment and compliance, the length of the clinic visits, the acceptability of the muffins and outcome measures, and the financial aspects and adequacy of resources.

## Materials and methods

### Study design

**Figure 1.**
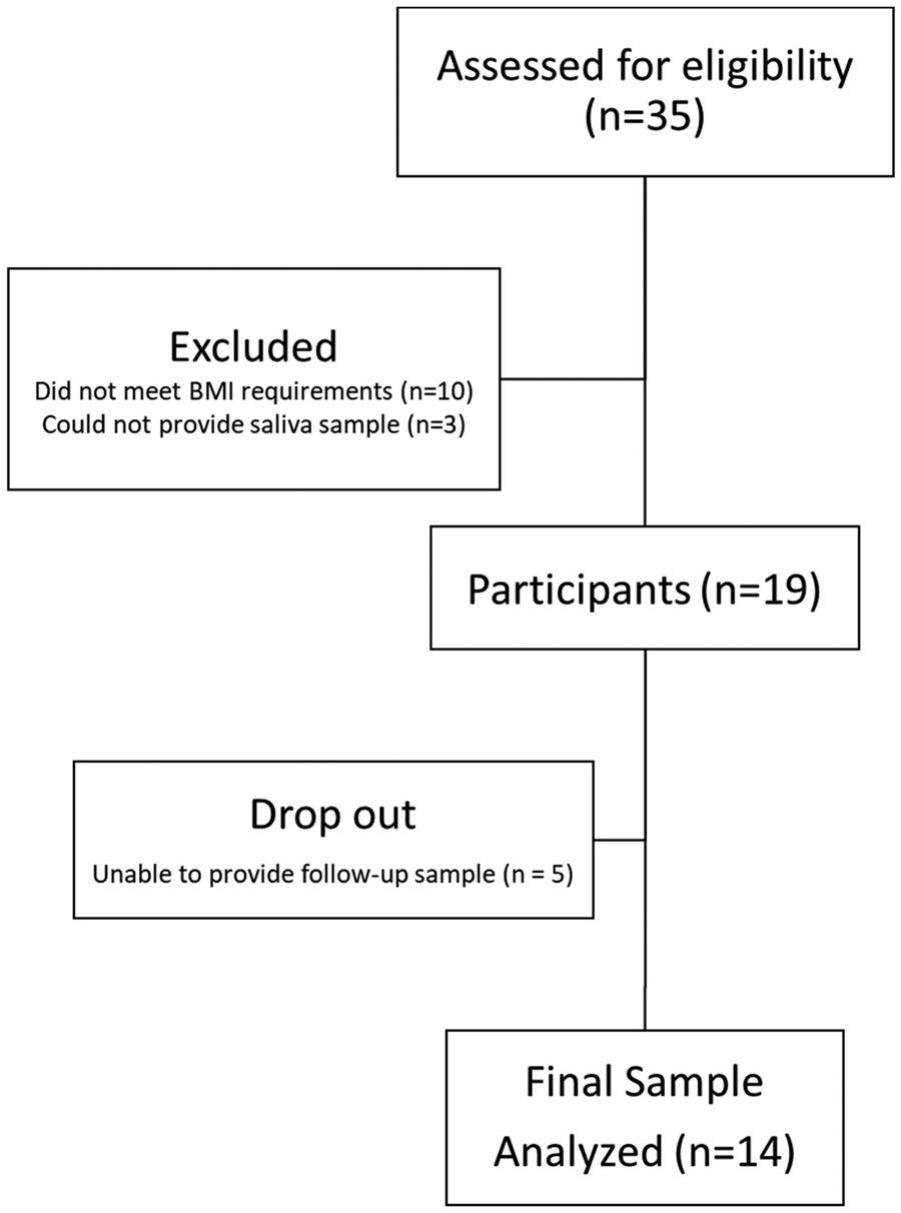
CONSORT Flow diagram.

A 10-day pre/post feasibility trial was conducted at the University of Florida. All participants provided their informed consent before initiation of the trial. The trial consisted of two clinical visits that were separated by 11 days. During the two visits, participants were provided with a baseline urine sample, 2 mL saliva sample, completed dietary and spice habit questionnaires, and then were provided with a 10-day supply of muffins that were frozen with instructions of how to consume. The study followed CONSORT guidelines for study design (Figure 1). This study was approved as exempt by the University of Florida IRB (IRB202101532) and registered on clinical trials (NCT05055362) in August 2021.

### Sample preparation

The muffin was developed in a food-grade facility at the University with a total weight of 60 g. Each muffin was prepared with 3 g turmeric (McCormick®) and 1 g cinnamon (McCormick®), 23.8 g unsweetened Milked Almonds™ (generously provided by Elmhurst®), 15 g whole-wheat flour (generously provided by Ardent Mills), 15 g honey (Glory Bee Pure Clover), 1.1 g Canola oil (Publix®), 0.5 g baking powder (Clabber Girl®), 0.4 g vanilla (McCormick®), 0.4 apple cider vinegar (Publix®), and 0.2 g baking soda (Arm&Hammer™). Based on the analysis through the Elizabeth Stewart Hands and Associates (ESHA) database, the nutritional composition of these muffins was 131 calories, 27 g total carbohydrates, 3 g protein, 146 mg sodium, 100 mg potassium, 73 mg phosphorus, and 50 mg calcium [31]. Moisture and water activity were measured to assess the safety of the product. They were determined at days 0, 7 and 10 for this muffin at 20 °C, −4 °C and −18 °C that resulted in acceptable levels for both tests.

A sensory evaluation among healthy adults (*n* = 10) was conducted prior to the initiation of the feasibility trial regarding overall acceptability, appearance, texture, and taste, and a question was included about length of days that they would consume the product with the options <7 days; 10 days; 14 days; or 29 days. All participants chose 10 days of consuming the product and indicated that the muffin was acceptable and that they enjoyed the taste. Feedback was given on improving the texture and durability of the muffin after three days. These same participants were then asked to participate in a second round of testing that consisted of storing three samples of muffins at a commercial freezer and consuming one a day for up to three days. Instructions were to thaw these muffins in a microwave at 100% power for 30 s, which all participants indicated was acceptable.

### Participants

Adults (>18 years old) who were obese (BMI > 24.9 kg/m^2^) without a known of chronic diseases were eligible to participate. Other inclusion criteria included no food allergies or dietary restrictions, no contraindications to consuming anything by mouth as per a medical professional and/or personal reporting and being able to provide two saliva samples. The selection of adults who were considered obese was based on evidence that this population has elevated levels of salivary inflammation markers [32,33]. Exclusion criteria included not meeting the above inclusion criteria as well as being pregnant/lactating. A urine sample was used to detect pregnancy.

Participants were recruited through advertisement with flyers in UF equal access clinics (*n* = 4), local family medicine clinics (*n* = 6), campus facilities (CALS, Gator Dining), Oak Hammock, UF health study listings, Food Science and Human Nutrition listserv email to students and faculty, CALS listserv to faculty and students, UF at work newsletter, study connect and Consent2Share with the assistance of the Clinical and Translational Science Institute (CTSI) recruitment center. Interested participants were screened *via* telephone to determine eligibility.

### Study procedure

Participants who met the inclusion criteria came to the clinical lab at University of Florida for completion of the study protocol at pre-assigned times. At day 0, all participants signed the informed consent form, a 2-ml saliva sample was taken 2 h post consumption of food and beverages other than water. A 50-ml first void urine sample was also collected to determine total protein, albumin to creatinine ratio (ACR), and pH. Saliva and urine samples were stored at −40 °C until further analysis. Participants’ height was measured using the stadiometer while weight was measured using the Omron® body composition scale (model HBF-514C). Participants were instructed to wear light clothing and to remove their shoes prior to stepping onto the scale. The scale was placed on a cement floor and calibrated prior to first participant use. Weights were taken twice to the nearest tenth in kg and then averaged for a score. Body mass index (BMI) was calculated using height in meters and weight in kg and categorized through the Centers for Disease Control and Prevention standards [34].

Participants completed a 30-day spice and semi-quantitative food frequency questionnaire (FFQ) and then received a 10-day supply of the muffins. Participants were instructed to consume this muffin daily for 10 days following the clinic visit with or without a meal, to store these muffins in a commercial freezer at −32 °C to preserve freshness, and to heat the muffins at 100% power in a microwave for 30 s, and to proceed with their typical dietary and spice habits. On day 11, participants returned to the clinic to provide a 2-ml saliva sample for 2 h post-consumption of foods/beverages other than water. They also completed a short yes/no survey if spice consumption or dietary habits changed during the trial and were asked questions about the acceptability of the muffins. Participants received a total of $10, $5 for each saliva sample provided.

### Participant compliance

Compliance was measured through a 10-day consumption log that was provided to participants at the first visit. Participants were instructed to fill it out every day and to estimate the percentage of the muffin they consumed. The consumption log had pictures and percentages for reference. They were also asked to state if they ate it with a meal or with any condiments, jams, jelly, or butter. Prior to the second study visit, participants were reminded to bring in their consumption log. Additionally, they received daily text messages from the researcher reminding them to consume their muffin, and further asked the amount consumed the previous day, and if they had any issues while consuming the muffins.

### Food frequency questionnaire

Participants responded to a modified version of Dana Farber’s Cancer Institute Eating Habits Questionnaire [35]. This questionnaire included five food categories—dairy, fruits, vegetables, meats, sweets, baked goods, and beverages—with a total of 61 food/beverage items based on the 9 frequency choices of never to 6 or more servings per day of consuming those items over the past year. The instrument was modified to remove an item from the vegetable category and an item from the meats, sweets, and baked goods due to redundancy, and asked participants about their consumption habits over the past 30 days as opposed to a year to reduce potential recall bias [36,37]. Participants indicated the frequency that they consumed these items and scored accordingly from never or less than once per month (0), 1–3 times per month (0.7), 1 time per week (0.14), 2–4 times per week (0.79), 1 time per day [1], 2–3 times per day (2.5), 4–5 times per day (4.5), 6+ times per day [6]. Based on the frequency that a participant indicated that they consumed the food/beverage, a score was provided adhering to the protocol by the EPIC food frequency questionnaire to determine energy and nutrient intakes [38].

### Spice consumption questionnaire

Participants responded to a validated 50-item questionnaire regarding their frequency and amount of 25 spices consumed over the past 30-days from never or less than once per month, 1–3 times per month, 1 time per week, 2–4 times per week, 1 time per day, 2–3 times per day, 4–5 times per day, and 6+ times per day [39]. Based on the frequency that a participant indicated that they consumed a spice, a score was provided adhering to the protocol by the International EPIC centers to determine total quantity consumed [38]. To determine the number of spices used when cooking or adding to a prepared dish, responses included never, do not measure, less than 1 teaspoon, one teaspoon, and more than one teaspoon. From these responses, a total score was provided from a minimum score of 0, showing no spice use to a maximum spice use of 37.5 teaspoons weekly. To determine the total amount of each individual spice consumed, the total teaspoon amount consumed was converted to mgs.

### Specimen collection and assay methods for urinalysis

Participants were instructed to avoid consuming anything but water 2-hours prior to the collection of urine and saliva samples. At least 50 ml first void urine samples were collected at day 0. Urine was thawed in a sonicator and brought to room temperature, 21 °C, upon analysis. From there, both strips were submerged in the urine and placed in their specific testing machines Multistix 10SG and Clinitek Microalbumin 2 for analysis [40]. ACR was measured with the Clinitek Status + analyzer and dip strips, while urinary protein and pH were measured with the Clinitek Advantus analyzer and dip strips (Clinitek Status + and Clinitek Advantus, Siemens, United States).

### Specimen collection and assay methods for inflammation markers

The saliva samples to detect inflammation markers, C-reactive protein (CRP) and interleukin-6 (IL-6), were initially centrifuged at 4000 rpm at 4° C for 20 min and measured using the ELISA

(Enzyme-linked immunosorbent assays) immunological tests.

#### CRP ELISA

CRP in saliva was measured at day 0 before the start of the study and at day 11 using Salimetrics’ salivary CRP ELISA kit (State College, PA, USA) according to the manufacturer’s instruction. Once plate layout was determined, the saliva sample was diluted x10 in the CRP sample diluent (15 µL saliva to 135 µL of CRP Sample Diluent) supplied by the kit. All samples were analyzed in duplicates. The plate was read at 450 nm, average optical density was computed for all duplicates and the calculated concentrations of the saliva samples were multiplied by the dilution factor of 10 to obtain final CRP concentrations in pg/mL.

#### IL-6 ELISA

IL-6 in saliva was measured at day 0 before the start of the study and at day 11 using Salimetrics’ salivary IL-6 ELISA kit (State College, PA, USA) according to the manufacturer’s instruction. Once plate layout was determined, the saliva sample was diluted x5 in the IL-6 sample diluent (60 µL saliva to 240 µL of CRP Sample Diluent). All samples were analyzed in duplicates. The plate was read at 450 nm, average optical density was computed for all duplicates and the calculated concentrations of the saliva samples were multiplied by the dilution factor of 5 to obtain final IL-6 concentrations in pg/mL.

### Statistical analysis

Statistical analyses were performed using SAS v9.4. Frequency and counts were reported for demographics, dietary and spice consumption. Changes in the primary outcome, salivary inflammation markers, were assessed by one-sample pre/post t-test. Significance was established at *p* < 0.05.

## Results

A total of 14 adults, who were predominately female (*n* = 10), an average age of 44 years, with a nearly equal distribution of races/ethnicities and who had an average BMI >32.16 kg/m^2^ completed the entirety of this trial. Twelve of the 14 participants had microalbumin levels (<30mg/L) and ACR (<30mg/g) that were within normal range [41], and all participants had varying levels of creatinine between 50 and 300 mg, all within normal range [42]. Two participants had microalbumin levels >30mg/L and ACR between 30–300 mg/g that were considered abnormal. pH levels varied between 5–7 and two participants had protein that was detected in the urine. Demographic information is summarized in Table 1.

**Table 1.** Participant demographics (*n* = 14).

Characteristics	N
Age (y in ranges)	44. 21 (18-70)
Female participants, n	10
Race/Ethnicity, n	
African American	2
Asian	3
Caucasian	4
Hispanic	5
Native American	1
Education level, n	
Some college credit, no degree	3
Associate degree	1
Bachelor’s degree	7
Master’s degree	2
Doctorate degree	1
BMI (kg/m^2^ range)	32.16 (26-43)
uACR (<30mg/g reference value)	<30 (12 of 14 participants) 30-300 (2 of 14 participants)
pH (4.6-8 reference value)	6.2 (14 participants)
Protein (negative reference value)	Normal (12 of 14 participants) Abnormal (2 of 14 participants)

### Evaluation of the feasibility of the pre/post-trial

#### Participant recruitment

Recruitment for this study was for a total of four months (September – December 2021) with participant enrollment occurring on an ongoing basis. Recruitment efforts started by placing flyers in equal access clinics and UF affiliated family medicine clinics. Additionally, flyers were posted around the UF main campus. After two weeks with a low response rate (<2 individuals), the flyer was sent out through listservs in UF, specifically UF health study listings, Food Science and Human Nutrition, CALS, UF at work newsletter, study connect. Additionally, the study was posted in Consent2Share with the assistance of the Clinical and Translational Science Institute (CTSI) recruitment center. A total of 35 prospective participants were screened with 19 agreeing and meeting the inclusion criteria. A total of 5 participants dropped with 3 unable to produce a second saliva sample, 1 did not provide a reason and 1 dropped due to illness unrelated to the muffins (Figure 1).

#### Participant compliance

At the second visit, only three participants filled out the consumption log with the remaining participants indicating that they consumed the entirety of the muffin. As no participant responded to the research coordinator’s text messages about the taste of the muffin or how they consumed the muffin (e.g., butter, with a meal, etc.) daily, at the second visit, these questions were asked. Participants had no adverse reactions or gastrointestinal issues with consuming the muffins. They consumed the muffins without adding condiments, butter and/or jellies/jams and normally at breakfast with just a beverage (e.g., coffee or tea). Half of the participants shared that after day 5, even though they followed the directions in heating the muffins, they had limited flavor as the previous days and only consumed them due to the trial.

Even though only 14 participants completed this study, reducing burden on participants by providing them with a 10-day supply of the muffins that they could consume at their place of convenience, allowing them to consume their typical dietary habits, and minimizing collection of information through two visits to collect a urine sample, two saliva samples and completion of questionnaires may have allowed for compliance with consuming these muffins and remaining in the study.

#### Sample collection

The process for collecting the markers and the self-assessment of dietary and spice habits was appropriate for obtaining the data. Each visit to the clinic was about an hour, which was adequate time to collect the information and for participants to share their experiences. No participants missed visit times or overlapped with other participants. For the saliva samples, as many were experienced with providing them for COVID-19 checks, the longest time to produce 2mls was about 8 min with the shortest time being 1 min. Of the five participants who dropped from the study, three did due to the inability to produce a second saliva sample. The researchers used techniques that were presented in the instructions of the saliva collection kit but possibly other techniques to help stimulate saliva are warranted.

The dietary and spice habit questionnaires were administered paper-based, and participants completed them while at the visit. Each questionnaire took no longer than 10 min and no participant indicated it was preferable online.

The lack of results for the saliva CRP marker necessitates modification to the trial and/or the outcome measure prior to expanding to larger randomized trials. The consumption of muffin with 3 g of turmeric, which demonstrated a statistical difference in saliva IL-6 and a decreasing trend in CRP, illustrates that a lengthier trial may aid in a more significant finding. The assays produced results in range, thus the dilution factor and the tests were performed correctly.

Resources were appropriately budgeted and planned as illustrated by the completion of the feasibility trial. Even though the recruitment extended beyond the projected date, the analysis of data was within an appropriate time to completion. For a larger randomized control trial, it is acceptable to purchase ELISA kits post-enrollment to ensure minimal waste as kits expire within 12 months.

Sample sizes for a randomized controlled trial were calculated using the outcome measures obtained in this feasibility trial. For IL-6, results showed that a total of 27 participants would be needed to detect a statistically significant effect of the muffins with turmeric at an alpha of 0.5 with an 80% power. For CRP, results showed that a total of 76 participants would be needed to detect a statistically significant effect at an alpha of 0.5 with an 80% power.

### Inflammation biomarkers results

**Figure 2.**
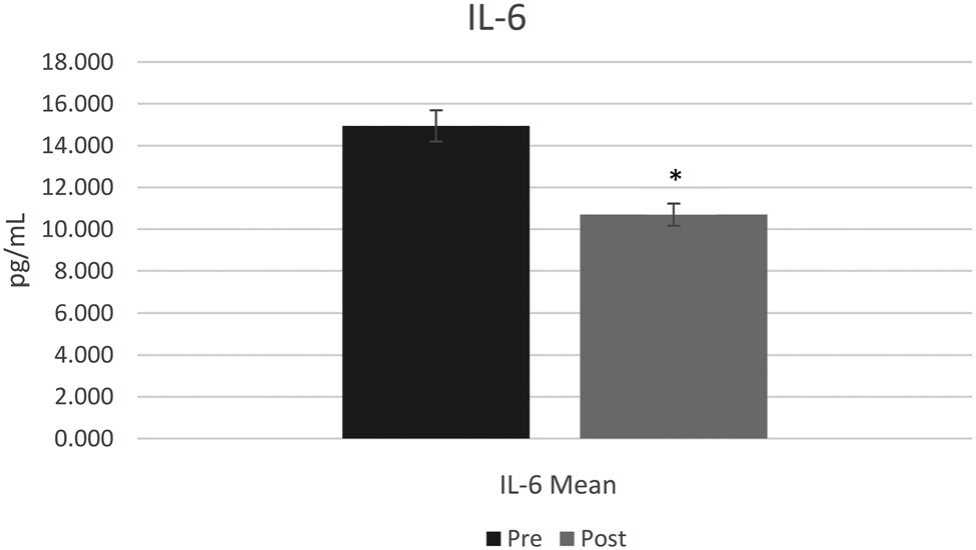
Interleukin-6 means pre and post for all participants (*n* = 14). Pre-Interleukin-6 mean was 14.9 ± 11.6 and post interleukin-6 mean was 10.6 ± 6.7. *=(*p* < 0.05).

**Figure 3.**
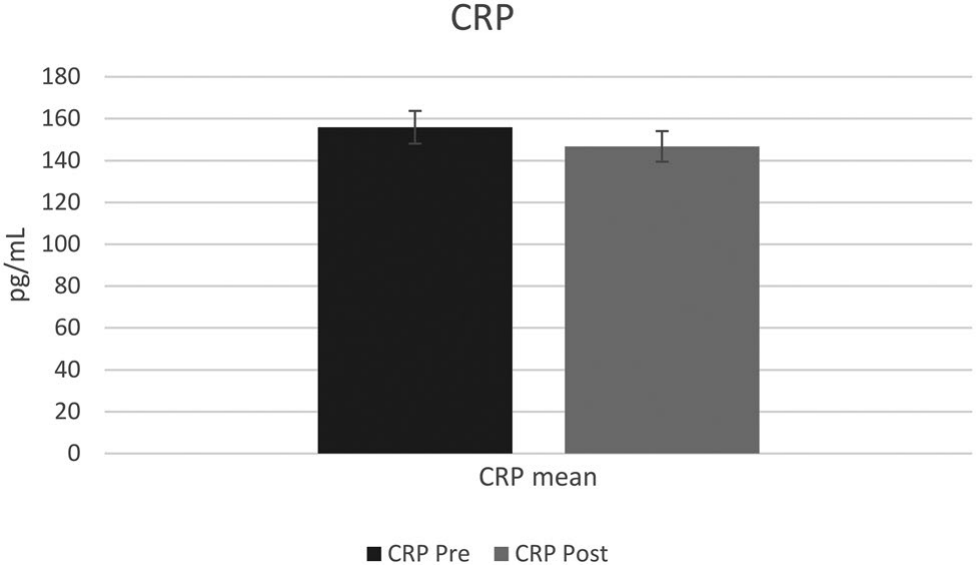
C-reactive protein means pre and post for all participants (*n* = 14). Pre C-reactive protein mean was 155.8 ± 142.8 and post C-reactive protein mean was 155.8 ± 131.1. (*p* > 0.05).

A t-test with a constant sample resulted in a significant decrease in saliva IL-6 post-trial (mean difference = 4.25, sd = 6.41, *p* = 0.03) (Figure 2) with no statistical difference observed in saliva CRP post-trial (mean difference = 62, sd = 43, *p* > 0.05) (Figure 3).

### Food and spice frequency

On average, most participants consumed fruits once daily, ranging from 1–3 times monthly up to 3–4 times daily. Participants, on average, consumed vegetables once or twice daily, ranging from once monthly up to five times daily. As for dairy products, the cheese and butter were consumed most frequently at 5–6 times weekly or 2–3 times weekly, respectively. Twelve of the 14 participants ate chicken and eggs at least once per week, with an average of 5–6 times weekly. Beef, pork, and fish were consumed about once per week on average. Cookies and chocolate were also common, with an average of 1–2 times weekly, while cake, pies and candy were only consumed once monthly. There was an even consumption of white and dark bread, with an average consumption of twice weekly. As for beverages, most participants had coffee at least once a day, alcoholic beverages were consumed once weekly, and low-calorie soda 1–2 times weekly, on average. From the 10-day trial, no participants changed dietary habits. Table 2 includes the average consumption of energy and nutrient intake based on frequency of foods/beverages consumed.

**Table 2.** Average consumption of energy and nutrients of participants (*n* = 14).

	Mean (SD)
Energy (kcal)	1662.8 (919)
Carbohydrate (g)	104.9 (85.97)
Protein (g)	69.85 (55.60)
Total fat (g)	79.3 (46.83)
Vitamin E (mg)	5.5 (3.48)
Vitamin C (mg)	48.7(32.9)
Magnesium (mg)	165.5 (113.4)
Omega-3 FA (µg)	1.0 (0.7)
B-carotene (µg)	1.7 (1.4)

For the spice consumption, participants consumed, on average, a range of at least 1 tsp once weekly to up to 5 times weekly of certain spices, two participants consumed spices once every two weeks, while five participants did not consume or prepare meals with any of the listed spices. Further analysis of total consumption of each of the eight spices showed that participants consumed on average 2.27 mg of garlic and 1.68 mg of black pepper weekly. All other spices included in the instrument were consumed negligibly (Table 3).

**Table 3.** Average spice consumption for participants (*n* = 14).

Spice	Mean (SD)
Black pepper (mg)	1.68 (1.72)
Chili pepper (mg)	0.49 (0.84)
Cinnamon (mg)	0.58 (0.7)
Cumin (mg)	0.34 (0.56)
Garlic (mg)	2.27 (3.25)
Ginger (mg)	0.34 (0.52)
Paprika (mg)	0.23 (0.43)
Turmeric (mg)	0.44 (0.69)

## Discussion

This 10-day pre/post feasibility trial assessed the influence consumption of a muffin with turmeric had on salivary CRP and IL-6 in adults who were overweight/obese. Overall, there was a significant reduction in saliva IL-6 (*p* < 0.05) with none observed in CRP (*p* > 0.05). Frequency of dietary habits and spice intake remained similar over these 10 days.

Obesity is considered a low-grade chronically inflamed state that presents higher levels of IL-6 and CRP in the body [43,44]. The results of this study support the previous literature indicating that spices such as turmeric, specifically its bioactive compound curcumin, can decrease inflammation markers such as IL-6 [22,45,46]. In this study, the significant reduction in IL-6 (*p* < 0.05) may be explained by the regulation of the nuclear factor kappa-B (NFkB) pathway. IL-6 is a pro-factorial cytokine that induces the expression at the transcriptional level of genes with critical roles in inflammation [47] and is mediated by the NFκB inflammatory signaling pathway that helps regulate the expression of IL-1β, IL-6, TNF-α, COX-2 and prostaglandin E2 (PGE2) [48]. This pathway is the principal mediator of systematic inflammatory responses, as is the case in chronic diseases and obesity [48]. In this pathway, curcumin regulates NFkB expression by reducing phosphorylation and degradation of IkBa, a known precursor of NF-kB [21], which by a reaction cascade leads to lower IL-6 expression. Even though this study did not explore the mechanism by which IL-6 was reduced, previous studies explored the effect of turmeric both in IL-6 serum markers and in NF-kB mRNA expression. A post-hoc analysis of a randomized controlled trial measured the effect of supplementation with turmeric supplements on inflammation in adults with metabolic syndrome (*n* = 117) [49,50]. Participants were randomized to receive turmeric supplements (1000 mg) with a co-supplementation of piperine to boost bioavailability or placebo for 8 weeks. Results showed significant reduction in serum concentrations of TNF-α, IL-6, TGF-β and MCP-1 in the turmeric versus placebo group (*p* < 0.001). Another randomized double-blind controlled study looked at the impact of curcumin supplementation on expression of inflammatory transcription factors, specifically qPCR of NF-ƙB, of adults on hemodialysis (*n* = 28) [46]. Participants consumed carrot juice with curcumin, at 95% in 2500 mg turmeric, 3 times weekly at the end of the dialysis session for 12 weeks. The results showed a significant decrease in NF-kB mRNA expression for the curcumin group (*p* < 0.05), which was not present in the control group. These studies support the conclusion that curcumin influences IL-6 through the NF-kB pathway, reducing inflammation in adults with metabolic syndrome and on dialysis.

Few studies, though, have focused on spices mixed with whole foods. Blanton and Gordon conducted a feasibility study with obese adults (*n* = 4) to determine the impact an egg infused with 5000 mg of turmeric on urine oxidative stress markers over 6-hours. Results showed no differences in the stress markers over this period, which may have been attributed to the timing or type of markers assessed per the researchers [51]. Moreover, a 4-week randomized crossover trial with healthy overweight men (*n* = 6) determined if the incorporation of spices into the diet can help enhance antioxidant defenses [52]. Participants were given either a control meal or a meal with 14 g of a spice mixture that included turmeric (1000 mg) during two different visits to the clinic, with a 1-week separation between visits, taking blood samples before and after each meal. The study showed that adding spices to the meal significantly increased the ferric reducing antioxidant power, showing that antioxidant capacity was higher following the spiced meal compared to the control.

In contrast with the previous studies, this present trial used 3000 mg of turmeric powder, which contained 200 mg of curcumin, a dose similar to other studies that have used between 30 mg to 2000 mg of curcumin in a capsule among an obese population [53–55]. However, it is important to note that the curcumin amount in turmeric powder can vary significantly between treatments [56]. A quantitative study compared the amounts of curcumin present in several brands of turmeric and curry powder using HPLC. Turmeric powders had 3.14% curcumin by weight on average, whereas curry powders had less than 1% on average, with great variability [56]. Another important factor regarding dose is curcumin’s bioavailability once ingested. The use of fat is known to increase bioavailability and absorption of curcumin [57], thus, in the composition of the muffin in this study, 1 g of canola oil was used. Lastly, cinnamon and honey were added for flavor, but the quantities added (1 g cinnamon and 15 g honey), were below the quantities established for these compounds to influence inflammation [58,59].

In the case of CRP, results showed a decreased trend without statistical significance. One hypothesis that explains these results is that IL-6 induces transcription of the CRP gene through activating the STAT3 transcription factor [60,61]. Since regulation of CRP by IL-6 happens at the transcription level, the researchers hypothesized that a longer study duration might be necessary to influence CRP levels. This hypothesis is based off other clinical trials where turmeric supplementation significantly reduced serum CRP levels [46,62]. A double-blind randomized controlled trial conducted on adult males with metabolic syndrome (*n* = 250) found that turmeric supplementation caused a significant reduction in serum CRP after 8 weeks of treatment [62]. The patients received either back pepper (1500 mg/d), turmeric (2400 mg/d), a combination of both black pepper and turmeric (900 mg black pepper and 1500 mg turmeric), or placebo. Results showed that the combination group had significantly lower CRP serum levels compared to placebo (*p* < 0.001) and to baseline levels in the same group (*p* < 0.01). The same randomized controlled trial that was previously mentioned for its decrease in NF-kB also showed a decrease in hs-CRP after 12 weeks of treatment [46]. Lastly, a systematic review and meta-analysis of randomized controlled trials measured the effect of turmeric on inflammatory markers in chronic diseases and showed that serum CRP levels were significantly reduced in the curcumin supplementation groups. However, all these trials lasted more than 10 days, ranging from 4 weeks to 4 months [54].

The duration of the study is a factor to highlight when discussing the results. Previous meal studies with spices focused on postprandial effect on inflammation/oxidative stress responses within a 6–8-hour period and supplementation studies lasting more than 4 weeks. Due to the feasibility nature of this study, as it is currently not known the half-life of salivary IL-6 and CRP, it was conceived that CRP levels double every 8 h and peak 42 h after initial stimulus and that circulating IL-6 has a half-life of 15.5 h [63,64], thus, it was necessary to assess the influence turmeric on these markers over several days. One of the main aspects of this study was providing turmeric easily and collecting markers at low-cost. Previous studies that have included turmeric as part of the food instead of as a supplement have done so with an extensive diet, breaking up the mgs of turmeric into different meals. Although, with this approach, compliance is difficult to measure, and it might not represent a realistic solution for many adults [19,52]. For this study, a food was developed that was easy to consume and not bothersome to daily routine.

An important limitation to take into consideration with human trials is the complexity of participant recruitment and compliance. Recruitment for this study was initially planned to be executed in one month, with all or at least most participants participating in the study at the same time. However, recruitment spanned over four months, with recruitment strategies being modified to adapt to the target population. Some strategies involved in recruitment were to post flyers in family medicine clinics and medical spaces that would allow it, going in person to recruit participants at these clinics, and posting the study on listservs. Through this study, the most effective recruitment strategy was UF health study listings. This method of recruitment sends out blast emails to those individuals who have registered to receive information about upcoming or on-going studies that they may be qualified to participate in. As the intention of this trial was to recruit and start the trial at one time with all participants, due to limited interest when recruitment began, the trial transitioned to rolling basis. This proved to be a challenge in the scheduling of participants to attend the clinic and to ensure proper communication was occurring between the research coordinator and the participants at different times of the trial. Lastly, even with the plans put in place to measure compliance, it was still not completely effective because of the self-assessment strategies (e.g., amount of muffin consumed, how it was consumed with or without products). Some strategies that were implemented to ensure transparency was explaining the importance to ensure accurate results and to aid in designing studies that resulted in improvements in health outcomes and that lack of compliance would not affect the participant remuneration. Some other strategies that could be implemented would be to provide participants with containers to place any remaining food for the research team to measure.

Overall, this is the first trial that focused on the impact turmeric in a baked product had on salivary CRP and IL-6. Results showed a decrease in these markers with one being significantly decreased; however, some limitations must be highlighted. When assessing inflammation with different biomarkers, it is necessary to know the specificity and validity of each biomarker. A study that looked at the role of IL-6 in inflammation concluded that IL-6 plays three kinds of roles in inflammation - pro-inflammatory, anti-inflammatory, or repair-oriented [6]. Given this information, measurements of IL-6 might provide information about any of these three without being able to confirm which one [6]. This would present a significant limitation in the study since those were the only ways by which inflammation was measured. For future studies, a more holistic approach should be taken for measuring inflammation of participants.

Another limitation was that the biomarkers were measured through saliva instead of blood. Research on the use of saliva to measure IL-6 and CRP is emerging and some studies show that saliva testing does not have the same accuracy and validity as does blood, but saliva testing is not detrimental to the results [65]. CRP and IL-6’s half-life in blood is 19 h and 15 h, respectively. However, their half-life in saliva is yet to be determined and this might present a limitation when measuring inflammation through these markers. Thus, future studies should focus on assessing both saliva and blood for more accurate information about the impact spices and other dietary components have on inflammation. The sample size of 14 can also be seen as a limitation for this study. This sample was sufficient for a feasibility study, but it is difficult to generalize the results to the average population without doing stratified analysis that requires larger samples.

Even though this study was for 10 days, the results showed that turmeric in a muffin can lower salivary inflammation markers. A longer study with a randomized design must now be conducted to truly investigate the effect of curcumin in inflammation. Additionally, the inclusion of food frequency questionnaires is of great importance to explore any cofounding variables that might come into play. In future studies, participants should provide three 24-hour dietary recalls instead of a food frequency questionnaire to better assess their specific eating patterns in the period of testing.

## Data Availability

The authors confirm that the data supporting the findings of this study are available within the article.
